# Case Report and Literature Review: Clinical Characteristics of 10 Children With *Mycoplasma pneumoniae*-Induced Rash and Mucositis

**DOI:** 10.3389/fped.2022.823376

**Published:** 2022-03-03

**Authors:** Ning Chen, Miao Li

**Affiliations:** Department of Pediatrics, Shengjing Hospital of China Medical University, Shenyang, China

**Keywords:** *Mycoplasma pneumoniae*, pneumonia, MIRM, Stevens-Johnson syndrome, glucocorticoids, IVIG, children

## Abstract

*Mycoplasma pneumoniae*-induced rash and mucositis (MIRM) is a rare disease, which has not been reported in northern China previously. We retrospectively analyzed the clinical characteristics, diagnosis and treatment of 10 cases of MIRM in order to help clinicians to identify MIRM and to distinguish it from the similar mucositis and cutaneous characteristics of Stevens-Johnson syndrome. All 10 children included in the study had MIRM with skin and mucosal symptoms, but the characteristics of the skin and mucosal lesions differed by age. Most of the older children had sparse erythema and a vesicular rash, but the younger children had dense erythema without blisters but with purulent exudation. The mucositis was relatively mild in the younger children. The erythrocyte sedimentation rate, the levels of C-reactive protein, lactate dehydrogenase, and D-dimer were significantly elevated in most children with MIRM. Concomitant treatment of glucocorticoids and/or IVIG with macrolides may shorten the duration of fever and accelerate the clinical recovery. Additional case reports are needed to improve knowledge of the characteristics of MIRM and its response to therapy.

## Introduction

*Mycoplasma pneum*oniae (MP) is one of the most common pathogens that cause community-acquired pneumonia (CAP). In northern China, *Mycoplasma pneumoniae pneumonia* (MPP) accounts for 37.5% of the cases of CAP in children (1). Most cases occur in patients older than 5 years, whereas infection in younger children tends to be milder (1, 2). In addition, approximately 25% of children with *MP* infection have extrapulmonary complications, such as myocarditis, hepatitis, encephalitis, thrombocytopenic purpura, autoimmune hemolysis, and skin and mucosal damage (2–4). *MP* is rarely isolated in non-pulmonary samples, which suggests that the extrapulmonary manifestations are due to the immune response to *MP* infection (2, 5, 6).

*Mycoplasma pneumoniae*-induced rash and mucositis (MIRM) is a rare disease, and is characterized by mucositis with prominent sparse vesiculobullous and/or target-like eruptions. Diagnostics criteria were proposed in 2015 (5), and have a good prognosis. The pathogenesis of MIRM differs from that of erythema multiforme, Stevens-Johnson syndrome (SJS) and toxic epidermal necrolysis (TEN), and although the rashes have similarities, the treatment is different. Identifying the characteristics of MIRM is a challenge for clinicians, and because of the low incidence rate of MIRM, clinicians are often confused by these similar diseases. To date, there have been no case reports of MIRM in northern China. We retrospectively analyzed the clinical characteristics, diagnosis, and treatment of MIRM in children admitted to our hospital, in order to summarize the clinical characteristics and treatment of MIRM in detail and to improve clinicians' ability to identify MIRM and to distinguish it from conditions with similar mucositis and cutaneous characteristic, such as SJS.

## Case Series

### Clinical Presentation

We conducted a retrospective analysis of 10 cases of children with MIRM, hospitalized with *MP* infection in the Department of Pediatric Respiratory Medicine at Shengjing Hospital in Shenyang. The cases were diagnosed according to diagnostic criteria proposed in 2015 (5), namely: (1) Distinct morphology with prominent mucositis and when cutaneous involvement was present with a characteristic sparse vesiculobullous and/or targetoid eruption; (2) Milder disease course with infrequent long-term sequelae and exceedingly rare mortality; and (3) Pathophysiology that was distinct from other erythema multiforme-spectrum diseases, including direct cutaneous infection. Children who taking oral antiepileptic drugs before hospitalization, had coinfections with other pathogens, were diagnosed with chronic eczema, other skin diseases, primary immune deficiencies, or autoimmune diseases, were excluded. A total of 18,730 children with *MP* infection were treated in the Pediatric Respiratory Medicine Department of Shengjing Hospital from January 2013 to December 2020. Only 10 patients (8 males and 2 females) were diagnosed with MIRM, and all cases were diagnosed by specialist dermatologists and ophthalmologists. The age of the patients ranged from 10 months to 11 years (median age: 7 years), and 3 patients were aged under 2 years (10 months, 15 months, and 24 months). Data were collected retrospectively on age, sex, duration of fever, duration of hospitalization, respiratory symptoms, the morphology of the mucocutaneous lesions, chest computed tomography (CT), treatment response and sequelae. The laboratory test results of the complete blood cell count, erythrocyte sedimentation rate (ESR), C-reactive protein (CRP), alanine aminotransferase (ALT), aspartate aminotransferase (AST), lactate dehydrogenase (LDH), and D-dimer level, and the antinuclear antibodies (ANA), antineutrophil cytoplasmic antibodies (ANCA), and *MP* immunoglobulin M (IgM) were analyzed.

**Figure 1:**
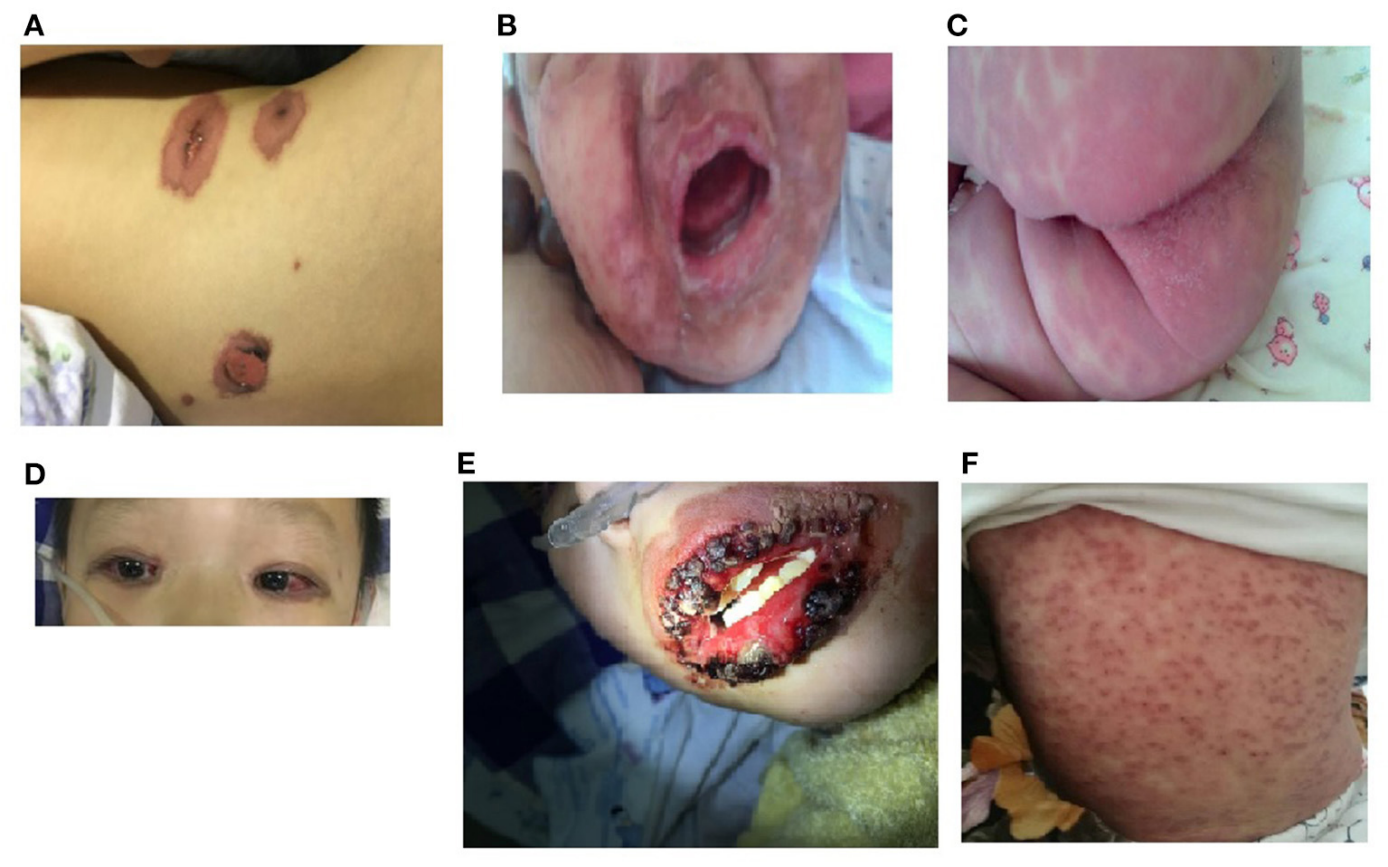
Morphological characteristics of rash of younger and older MIRM children. **(A)** An older child with a sparse red maculopapular rash, with some vesicular lesions. **(B,C)** Relatively mild mucositis in children aged <2 years, with the dense erythema without blistering, less purulent exudation, and no hemorrhagic lesions. **(D)** Conjunctival hyperemia of an older child's eyes. **(E)** Hemorrhagic crusting eruption on the lip of an older child. **(F)** Targetoid lesions and purpuric macules of the skin of a child aged <2 years old.

Because all the cases were selected from the respiratory medicine department, all 10 patients had obvious respiratory symptoms. They all had high fever and cough, 3 patients had wheezing, and 5 patients had hypoxemia. All patients had abnormalities on lung auscultation: 8 patients had moist rales in both lungs, and 2 patients had wheezing. Chest CT was performed in all of the patients, and showed lobar pneumoniae or segmental consolidation in 6 patients, necrotic pneumonia in 1 patient, the tree-bud sign and ground-glass opacities in both lungs in 4 patients. The fever, cough, and other respiratory symptoms appeared 2–11 days (median: 6.5 days) before the appearance of the mucocutaneous lesions, and 3 of the older children complained of eye discomfort 1–3 days before the appearance of the mucocutaneous lesions. All patients had skin lesions, with rashes distributed on the face, trunk, and limbs. The maculopapular rash in the 7 older children was sparse at the beginning, and then rapidly became blister in appearance, and 5 had fragile, easily broken blisters. The 3 children aged <2 years had maculopapular rashes without a herpetiform appearance, and 2 had target-like red maculopapular lesions with partial fusion, and purpuric macules (Figure 1). All patients had conjunctival hyperemia or ulcers, without corneal involvement. The 5 older children were more likely to have hemorrhagic secretions on their eyelid margins, and children aged <2 years were more likely to have purulent secretions. Nine children had painful multiple mucosal ulcerations on their buccal mucosa, palate and tongue, which caused difficulty with eating. The lips of the older patients tended to be swollen, with hemorrhagic crusting. However, the lips of the children <2 years old were normal in appearance, with less exudation. Seven patients had anal swelling, blisters or erosions; 6 patients had erythema, rashes and blisters on their genital organs; 4 patients had urinary meatus ulcers without urinary symptoms such as frequent urination, urinary urgency, or dysuria; 5 patients had nasal symptoms, 3 older children of them had thick bloody scabs in their noses, and 2 younger children of them had a purulent nasal discharge (Figure 1 and Table 1).

**Table 1:** Case descriptions of 10 patients with MIRM.

**Diagnostic criteria (5)**	**Age**	**Sex**	**Hospitalization time(d)**	**Detachment**	**NO. of mucosal sites involved**	**Few and fleeting morbiliform lesion, or few vesicles**	**Evidence of atypical pneumonia**	
							**Clinical (Fever, cough, positive auscultatory findings)**	**Laboratory (Increase in *MP* IgM antibodies, *MP* in oropharyngeal or bullae cultures or PCR, and/or serial cold agglutinins)**
Patient 1	5 y	M	11	<10% BSA	≥2 Oral ulcer, conjunctivitis	Sparse erythema, bullous lesions on the face, trunk, and limbs	Fever, cough, moist rales	*MP*-PCR (–), *MP*-IgM (+)
Patient 2	10 y	M	20	<10% BSA	≥2 Oral mucosa erosions, conjunctivitis, genital and anal erosion	Sparse bullous lesions on the face, trunk, and limbs	Fever, Cough, moist rales	*MP*-PCR (+), *MP*-IgM (+)
Patient 3	7 y	M	15	<10% BSA	≥2 Oral mucosa erosions, conjunctivitis, nasal, genital and anal erosions	Sparse Target lesions and blisters on the face, trunk, and limbs	Fever, Cough, moist rales	*MP*-PCR (+), *MP*-IgM (+)
Patient 4	7 y	F	14	<10% BSA	≥2 Oral mucosa erosions, conjunctivitis, nasal, genital and anal erosions	Sparse bullous lesions on the face, trunk, and limbs	Fever, Cough, moist rales	*MP*-PCR (+), *MP*-IgM(+)
Patient 5	9 y	M	10	<10% BSA	≥2 Oral and conjunctiva Hyperemia, anal erosions	Sparse bullous lesions on the face, trunk, and limbs	Fever, Cough, moist rales	*MP*-PCR (+), *MP*-IgM (+)
Patient 6	11 y	M	20	<10% BSA	≥2 Oral mucosa erosions, conjunctivitis, nasal, genital and anal erosions	Sparse bullous lesions on the face, trunk, and limbs	Fever, cough, moist rales, wheezing	*MP*-PCR (+), *MP*-IgM (+)
Patient 7	9 y	F	15	<10% BSA	≥2 Oral mucosa erosions, conjunctivitis, genital erosions	Sparse bullous lesions on the face, trunk, and limbs	Fever, Cough, moist rales	*MP*-PCR (+), *MP*-IgM (+)
Patient 8	10 m	M	11	25% BSA	≥2 Purulent conjunctivitis, purulent nasal discharge, anal swelling	Target lesions on the face, trunk, and limbs	Fever, Cough, wheezing	*MP*-PCR (+), *MP*-IgM (+)
Patient 9	15 m	M	13	>15% BSA	≥2 Oral ulcer, purulent conjunctivitis, purulent nasal discharge, genital and anal erosions	Maculopapular eruption, purpuric macules on the face, trunk, and limbs	Fever, Cough, wheezing	*MP*-PCR (+) *MP*-IgM (+)
Patient 10	24 m	M	19	30% BSA	≥2 Oral ulcer, conjunctivitis,	Erythema multiforme on the face, trunk, and limbs	Fever, Cough, moist rales	*MP*-PCR (+), *MP*-IgM (–)

*BSA, Body surface area*.

### Laboratory Investigations

*MP* infection was diagnosed based on a positive *MP*-immunoglobulin M (IgM) antibody test on serology, and a nasopharyngeal swab that tested positive for *MP*-DNA on polymerase chain reaction (PCR) testing. (*MP* antibody IgM antibody kit; Shenzhen PuRuiKang Biological Technology, Co., Ltd., Shenzhen, China) and *MP*-DNA identification (*MP*-DNA detection kit; Shenzhen PuRuiKang Biological Technology, Co., Ltd., Shenzhen, China). Samples were collected within 24 h after admission. An *MP*-IgM antibody result of > 1.1 signal-to-cutoff ratio (S/CO) was regarded as positive, < 0.8 S/CO was regarded as negative, a S/CO between 0.8 and 1.1 was regarded as weak negative. All of the patients had negative for blood cultures for bacteria, *respiratory syncytial virus* (RSV), *adenovirus* (ADV), *Epstein-Barr virus* (EBV), *influenza A virus, influenza B virus, herpes simplex virus* (HSV), and *Chlamydia pneumoniae* (CP). The laboratory test results of the 10 MIRM patients are shown in Table 2. The patients' ferritin, AST, blood urea nitrogen, and creatinine levels were normal, so some of these values are not listed.

**Table 2:** Laboratory test results of the 10 patients with MIRM.

**Laboratory examination (Normal value)**	**Patient 1**	**Patient 2**	**Patient 3**	**Patient 4**	**Patient 5**	**Patient 6**	**Patient 7**	**Patient 8**	**Patient 9**	**Patient 10**
WBC (× 10^9^/L) (3.9 ~ 9.7)	8.2	15.4	9.9	17.2	5.06	5.6	9.36	18.65	7.4	7.65
Neutrophils (%) (42.3–71.5)	70.2	80.5	80.2	70.3	74.3	81.4	62.6	69.9	63.9	39.54
Neutrophils (× 10^9^/L) (1.9–7.2)	5.76	12.4	7.2	12.2	3.8	4.7	5.9	13.0	4.73	3.02
Lymphocytes (%)(16.8–43.4)	20.8	8.9	7.9	13.2	7.0	11	27.2	15.1	22.9	53.08
Lymphocytes (× 10^9^/L)(1.1–2.7)	1.71	1.37	0.8	2.3	0.4	1	2.5	2.8	1.71	4.06
Eosinophils (%)(0.7–7.8)	2	2.6	0.3	2.4	0	0.1	0.1	0.3	3.4	1.93
Eosinophils (× 10^9^/L)(0.04–0.49)	0.16	0.4	0.32	0.41	0	0.01	0.01	0.06	0.25	0.15
Hemoglobin (g/l) (120~140)	127	136	130	132	135	127	134	112	101	103
Thrombocytes (× 10^9^/L) (135–350)	330	275	283	469	195	148	387	461	351	458
ESR (mm/l) (<20)	19	41	45	51	70	26	61	32	65	31
CRP(mg/dl) (0~8)	29.7	38.5	65.3	46	31.3	127	43.7	7.59	12.3	7.55
PCT(ng/ml) (<0.5)	0.05	<0.05	0.461	0.15	1.83	2.68	0.13	0.13	0.108	-
TP (g/l) (60–83)	69.8	67.1	67.5	69.2	80.3	67.5	69.9	72.6	65.5	48.2
Albumin (g/l)(35–53)	41.2	27	30.8	38.7	34.1	30.3	33.9	36.8	36.2	32.6
ALT(U/l) (0~40)	12	6	11	9	30	10	35	9	30	48
AST(U/l) (5~34)	23	15	16	23	36	19	28	26	39	21
LDH(U/l) (103–227)	313	405	198	489	576	362	275	308	422	416
T IgE (IU/ml) (<2 years; 0~12 IU/ml;2–4 years 0~33 IU/ml; 4–15 years> 85 IU/ml)	16.7	352.3	182	8.98	54.1	56.3	87.54	22.9	45.77	14.5
IgG (g/l) (4.82~12.2)	9.03	10.7	18.8	16.6	25.6	11.9	17.7	19.3	10.9	3.95
IgM (g/l) (0.41–1.65)	2.02	1.08	1.98	2.01	1.26	1.25	2.53	2.64	1.29	0.261
IgA (g/l) (0.42–1.58)	1.64	2.69	1.91	2.01	1.66	0.73	1.12	0.416	0.561	0.095
D-D (μg/L) (<252)	168	**-**	559	3,178	530	430	319	345	576	174
ANA	-	-	1:640	1:80	negative	-	1:80	-	negative	-
ANCA	-	-	negative	negative	negative	-	weak positive	-	positive	-

*ESR, erythrocyte sedimentation rate; CRP, C-reactive protein; PCT, procalcitonin; TP, total protein; ALT, alanine transaminase; AST, aspartate aminotransferase; LDH, lactate dehydrogenase; D-D, D-dimer; ANA, anti-nuclear antibody; ANCA, anti-neutrophil cytoplasmic antibody*.

### Therapy

All children were treated with macrolides azithromycin (10 mg/kg/d for 5 days) or erythromycin (30 mg/kg/d for 7 days) for *MP* infection. Ceftriaxone (40 mg/kg/qd) were also used in 5 cases because of bacterial co-infections. Nine patients received intravenous gamma globulin (IVIG) until the patient's body temperature was normal. Eight patients were started with intravenous methylprednisolone at an initial dose of 1–2 mg/kg per 12 h, after which their high fever alleviated quickly, and the dose of glucocorticoid was gradually reduced once the patient's temperature stabilized. The total duration of treatment ranged from 8 days to 4 months (median: 13 days). Generally, it took 1–4 days for the body temperature to return to normal after starting glucocorticoid therapy. No new rashes appeared after the patient's body temperature was stable for 2–8 days, and the mucositis and rashes gradually subsided. Because the oral mucosa recovers slowly, 5 patients still had pain and oral ulcers on discharge (Table 3).

**Table 3:** Treatment descriptions of the 10 patients with MIRM.

	**Patient 1**	**Patient 2**	**Patient 3**	**Patient 4**	**Patient 5**	**Patient 6**	**Patient 7**	**Patient 8**	**Patient 9**	**Patient 10**
Antibiotics (d)	Azithromycin (5 d)	Azithromycin (5 d) Cefuroxime (10 d)	Azithromycin (5 d) Ceftriaxone (13 d)	Azithromycin (5 d) Ceftriaxone (8 d)	Azithromycin (5 d)	Azithromycin (5 d) Ceftriaxone (11 d)	Azithromycin (5 d)	Erythromycin (11 d) Ceftriaxone (8 d)	Erythromycin (12 d)	Azithromycin (5 d)
Dosage and duration of IVIG treatment (d)	IVIG 400 mg/kg/d (5 d)	IVIG 300 mg/kg/d (5 d)	IVIG 400 mg/kg/d (5 d)	IVIG 400 mg/kg/d (3 d)	IVIG 200 mg/kg/d (5 d)	IVIG 400 mg/kg/d (3 d)	IVIG 200 mg/kg/d (5 d)	IVIG 200 mg/kg/d (3 d)	-	IVIG 400 mg/kg/d (5 d)
Dosage and duration of systemic methylprednisolone treatment (d)	1 mg/kg/q12 h ,× 7 d, then 1 mg/kg/d × 3 d (10 d)	2 mg/kg/q12 h × 7 d, then gradually reduced to stop (28 d)	1 mg/kg/q12 h × 6 d, then gradually reduced to stop (120 d)	2 mg/kg/q12 h × 5 d, then gradually reduced to stop (9 d)	2 mg/kg/q12 × 3 d, then 2 mg/kg/qd × 3 d, 1 mg/kg/qd × 2 d (8 d)	1 mg/kg/q12 × 10 d, then 1 mg/kg/qd × 5 d, and then 0.5 mg/kg/qd × 2 d (17 d)	1 mg/kg/q12 h × 10 d, then gradually reduced to stop (21 d)	1 mg/kg/q12 h × 6 d, then 1 mg/kg/qd × 3 d (9 d)	-	-
Fever duration before admission (d)	8	6	13	15	8	3	13	3	7	3
Fever duration after systemic methylprednisolone treatment (d)	1	2	1	1	1	3	1	2	-	-
Fever duration after IVIG treatment (d)	1	5	1	1	1	2	1	1	-	12
Total fever duration (d)	10	14	14	16	9	7	14	5	16	17
Duration of mucocutaneous lesions begin to subside after glucocorticoid treatment (d)	8	6	8	6	7	12	9	4	-	-
Duration of mucocutaneous lesions begin to subside after IVIG treatment (d)	8	9	8	6	7	10	9	3	-	18
Days of the rash begin to subside after admission (d)	9	12	8	6	7	12	9	4	13	19

“*-” No application*.

### Follow-Up

The titer of ANA antibody in Patient 3 was 1:640. In this case the laboratory test results of the ANCA, anti-Sm antibody, antiphospholipid antibody, and dsDNA were negative, complement 3 and complement 4, and renal and urinary function were normal. A rheumatic immunologist evaluated the patient and attributed the ANA result to *MP* infection. The child did not have symptoms of facial erythema or discoid lupus, so we excluded the diagnosis of systemic lupus erythematosus, and attributed the abnormal ANA to an immunological response to *MP* infection. We tried to discontinuing glucocorticoids several times, but the skin and mucosal symptoms reappeared whenever glucocorticoid was discontinued, so the patient was treated with glucocorticoids for 4 months. The patient's ANA antibody reverted to negative after 6 months (Table 3). At the one-year follow-up visit after discontinuing oral glucocorticoids, there were no abnormal clinical manifestations. Patient 9 refused immunotherapy, and his rash recurred several times over a 2-month period, with pigmentation.

One patient had an adhesion around the lateral canthus. One patient had a scar on the margin between the eyelid and eyelashes. The pulmonary ventilation function was normal in the 7 older children. All of the children were followed up for at least 6 months, and none developed chronic cough, wheezing, or restrictions with physical activity.

## Discussion

*MP* is a common pathogen that causes atypical pneumonia and respiratory tract infections in children, and can also lead to a variety of extrapulmonary complications. Approximately 22.7–25% of children with *MP* infection develop mucocutaneous complications (3, 6, 7), including urticaria, maculopapular rashes, erythema nodosum, Kawasaki disease, SJS, or TEN (3, 5, 6), but few cases meet the case definition of MIRM. Making the diagnosis is often difficult in patients with *MP* and rash and mucositis. In 2015, Canavan et al. (5) reviewed and classified MIRM as a new disease, and only 202 patients from 95 articles published between 1922 and 2013 were diagnosed with MIRM. The mucositis with or without skin damage caused by *MP* is relatively sparse, and distinguish from *HSV* and drug-induced SJS/TEN.

Reports of MIRM are relatively rare, and the incidence of MIRM may inaccurately report. We extracted the cases report of MIRM so far in Table 4, and summarized all the previous cases characteristics of MIRM reported in the medical literature. Within the past 8 years, only 10 cases of MIRM were diagnosed in our center, which was an incidence rate of 5.34/10,000 among children hospitalized with *MP*, which is considerably less than that previously reported (6, 13, 14). The majority of the patients (8/10, 80%) were male, as reported previously (5). Most of the cases occurred in older children, as reported previously (5). Although the age is an important characteristic of MIRM (15), MIRM can occur over a wide age-range, and has also been reported in adults (16, 17). There were 3 cases aged <2 years in our study, and the youngest case was aged only 10 months, which is rare. The incidence of MIRM is seasonal and is higher in the winter and spring, but can also occur in sporadic epidemics, such as in the epidemic of MMP reported by Watkins et al. (13).

**Table 4:** Summarizing all the previous cases characteristics of MIRM reported in the medical literature.

**Case reports**	**Age (years)**	**Sex**	**Fever**	**cough**	**lung lesions**	**Skin lesions**	**Mucosa lesion**					**Laboratory tests**	**Therapeutic aspects**	**Outcome**
						**Cutaneous**	**Oral**	**Ocular**	**Nose**	**Genital**	**Anal**			
Santos RP (8)	8	M	YES	YES	Atypical pneumonia	+	+	+		+		*MP* IgM (+)	Pain management, intravenous hydration and mucosal care, IVIGs	Noticeable clinical improvement
Li HO (9)	13	F	NO	YES	Bilateral streaking,	–	+	+	+	+		*MP* PCR (-)	Azithromycin cyclosporine A	Complete recovery
Li HO (9)	14	F	YES	YES	Dyspnea, expiratory wheezes	–	+	+		+		*MP* PCR(+)	Azithromycin cyclosporine A	Complete recovery
Li HO (9)	4	M	YES	YES	Atypical pneumonia	+	+	+				*MP* PCR(+)	Azithromycin cyclosporine A	No complications
Poddighe D (10)	10	M	YES	YES	Unknown	+	+			+	+	*MP* IgM (+)	Systemic steroids, clarithromycin	Complete clinical remission within a week
Meyer Sauteur PM (11)	12	M	YES	YES	Atypical pneumonia	+	+	+		+		*MP* IgM (+), *MP* PCR(+)	Doxycycline, methylprednisolone	Flagellate erythema on the anterior of the thorax at 3 months later
Bukhari EE (12)	12	M	YES	YES	Atypical pneumonia	+	+	+				*MP* IgM (+), MP PCR(+)	Antimicrobial therapy	Complete recovery

“*-” Without skin and mucosa lesions; “+” With skin and mucosa lesions*.

MIRM often involves 2 or more organs, including the eye, mouth, nose, digestive organs, or the genitourinary organs, and skin lesions may be mild or absent (5, 7, 18). The mucocutaneous lesions vary widely (5). All of the patients in our study had mucocutaneous eruptions, but the extent of the lesions varied by age. The majority of the older children showed sparse vesiculobullous lesions, which is consistent with a previous report (5). In contrast, younger patients often developed erythema or target-like lesions such as a herpetiform, generalized maculopapular rash. The conjunctiva (100%) and oral mucosa (90%) were always involved, and most patients had conjunctival congestion or ulcers. Many of the oral mucosal ulcers caused serious pain and difficulty with eating. Five older children had severe cutaneous erosions, with hemorrhagic crusting of the surface of the lips, eyelids, and nasal mucosa. The mucosal lesions of the younger children were relatively mild, with purulent exudation and purulent nasal secretions. The differences in the morphologies of the skin and mucosal lesions may due to the differences in the immune status of children of different ages. The lesions in urinary system are easily overlooked by clinicians because of the lack of urinary symptoms.

In this case study, all of the patients had respiratory symptoms due to *MP* infection. The CRP, ESR, LDH and D-dimer levels of the patients were all significantly higher than those of healthy children, and were diagnosed as refractory MPP (19). Clinical studies have reported that children with MPP that is accompanied by mucocutaneous lesions tend to have a longer duration of fever, longer hospitalization time, a higher CRP level, and are more likely to develop hypoxemia and other sequelae (6). This suggests that MIRM an extrapulmonary manifestation of refractory or severe MPP. Studies have found that serum total IgE levels in the patients with *MP*-associated extrapulmonary manifestations are higher than those in children with MPP alone (20). In this study, there were 5 patients with higher serum IgE levels than the correspondent upper limit of reference values for age, but none of them had a clear history of allergy. This increase of IgE level might reflect an immune imbalance after MP infection. It is easy for pediatricians with a limited understanding of MIRM, to misdiagnose it as a drug eruption or drug-induced SJS/TEN due to the patient's medication history of taking antipyretic drugs or cephalosporin before hospitalization. In addition, MIRM occurs mostly in children and young adults (5, 15, 18) with a better prognosis and low mortality (4, 21). Drug-related SJS (TEN) occurs mostly in adults and has a poor prognosis and high mortality (22). Because it is hard to tell from the clinical features, it requires a combination of pathogen detection and detailed medical history-taking in order to make the correct diagnosis. Moreover, MIRM is rarely associated with liver and kidney dysfunction and encephalopathy. Because some MIRM rashes are accompanied by blisters, they need to be distinguished from *HSV* infection. *HSV* infection generally causes a vesicular rash, which can occur on the trunk, limbs, lips, and in the mouth. In this study, the lips of the patients with MIRM often showed hemorrhagic crusting, but herpes labialis was rare, and all of the patients were negative for *HSV, CP* and other virus on isolation and culture, so we excluded other pathogen infection. These clinical characteristics provide clinicians with new ideas and perspectives for identification *MP*-associated mucocutaneous rashes so as to a proper treatment.

The pathogenesis of MIRM is unclear, but it is considered to be unrelated to macrolide resistance (13). A proposed pathogenic mechanism is that polyclonal B cells proliferate and produce specific antibodies and immune complexes after *MP* infection and are deposited in the skin, after which stimulated cytotoxic T-cells induce skin injury (5, 6, 23, 24). In addition, genetic susceptibility has been speculated to contribute to MIRM recurrence and family aggregation (5, 17, 24). In our study, Patient 3 had a high titer of ANA, which suggests that MIRM might induce inflammatory autoimmune disorders (4), so long-term follow-up is required.

As a newly recognized disease, there are currently no evidence-based guidelines for the clinical management of MIRM. MIRM is a self-limiting disease, so there is uncertainty about the optimal treatment. All of the patients with MIRM in our study received intravenous azithromycin or erythromycin therapy, nursing care of the skin and mucosa, and liquid diets, and 2 older children were given short-term parenteral nutrition due to pain with swallowing. Although the effectiveness of macrolides for *MP* is unclear, macrolides can reduce the amount of *MP* organisms in the airway and prevent transmission of *MP* (25, 26). Evidence on the effectiveness of immunotherapy for MIRM is limited; most treatment protocols are based on the SJS literature (5, 6, 27, 28), and evidence-based guidelines are lacking. From the perspective of the disease pathogenesis, the treatment of inflammatory cascade triggered by *MP* is more effective than antimicrobial therapy alone. The use of glucocorticoids in patients with macrolide-refractory MPP can significantly shorten the duration of fever and length of hospitalization, and inhibits the hyperinflammatory response (21, 29). IVIG can alleviate mucositis and other clinical symptoms (5, 28, 30, 31). Although there is a lack of consensus about the role of glucocorticoids in the treatment for SJS due to concern about the immunosuppressive effects, clinical studies have shown that glucocorticoids generally improve MIRM recovery (6, 21, 22). In this report, 8 patients were treated with a combination of methylprednisolone and IVIG for severe pneumonia and mucocutaneous lesions. This generally led to a rapid reduction in the high fever within 24 h, and alleviated their symptoms within the following 2–8 days. Children who were not given methylprednisolone, or who were given monotherapy with either methylprednisolone or IVIG, generally had a prolonged fever duration, and skin and mucosal lesions. Early use of cyclosporine may be an effective treatment for MIRM (9).

This study has some limitations. MIRM is a rare disease; the sample size is small, so the results may be biased. This study is a retrospective study, and some medical history in the patient records was not detailed, which may affect the conclusions of the study. It was a single-center study. As our center is a regional pediatric diagnosis and treatment center for northeastern China, most of the cases were severe, as milder cases are likely to have been treated in local hospitals or clinics, which may have led to the incidence being underestimated. Additionally, the abnormal ANA results in this study, which may not be representative of MIRM characteristics, so larger studies are needed to observe the change in ANA titers over the course of the disease and the effect of therapy. Therefore, we plan to carry out a multicenter prospective study to better understand the effect of treatment of MIRM.

## Conclusions

The clinical (cutaneous and mucosal) characteristics of MIRM can be heterogeneous and differ by age. Concomitant treatment of glucocorticoids and/or IVIG with macrolides may shorten the duration of fever and accelerate the clinical recovery.

## Data Availability Statement

The original contributions presented in the study are included in the article/supplementary material, further inquiries can be directed to the corresponding author.

## Ethics Statement

Written informed consent was obtained from the minor(s)' legal guardian/next of kin for the publication of any potentially identifiable images or data included in this article.

## Author Contributions

ML designed the study, coordinated and supervised data collection, and reviewed and revised the manuscript. NC collected data, drafted the initial manuscript, and reviewed and revised the manuscript. Both authors approved the final manuscript as submitted and agree to be accountable for all aspects of the work.

## Conflict of Interest

The authors declare that the research was conducted in the absence of any commercial or financial relationships that could be construed as a potential conflict of interest.

## Publisher's Note

All claims expressed in this article are solely those of the authors and do not necessarily represent those of their affiliated organizations, or those of the publisher, the editors and the reviewers. Any product that may be evaluated in this article, or claim that may be made by its manufacturer, is not guaranteed or endorsed by the publisher.

## References

[1] Gao LW Yin J Hu YH Liu XY Feng XL He JX. The epidemiology of paediatric Mycoplasma pneumoniae pneumoniae in North China: 2006 to 2016. Epidemiol Infect. (2019) 147:e192. 10.1017/S0950268819000839 31364532 PMC6518602

[2] Narita M. Pathogenesis of extrapulmonary manifestations of mycoplasma pneumoniae infection with special reference to pneumoniae. J Infect Chemother. (2010) 16:162–9. 10.1007/s10156-010-0044-X 20186455

[3] Narita M. Classification of Extrapulmonary Manifestations Due to Mycoplasma pneumoniae Infection on the Basis of Possible Pathogenesis. Front Microbiol. (2016) 7:23. 10.3389/fmicb.2016.00023 26858701 PMC4729911

[4] Poddighe D. Extra-pulmonary diseases related to Mycoplasma pneumoniae in children: recent insights into the pathogenesis. Curr Opin Rheumatol. (2018) 30:380–7. 10.1097/BOR.0000000000000494 29432224

[5] Canavan TN Mathes EF Frieden I Shinkai K. Mycoplasma pneumoniae- induced rash and mucositis as a syndrome distinct from Stevens-Johnson syndrome and erythema multiforme: a systematic review. J Am Acad Dermatol. (2015) 72:239–45. 10.1016/j.jaad.2014.06.026 25592340

[6] Meyer Sauteur PM Theiler M Buettcher M Seiler M Weibel L Berger C. Frequency and Clinical Presentation of Mucocutaneous Disease Due to Mycoplasma pneumoniae Infection in Children With Community-Acquired Pneumoniae. JAMA Dermatol. (2020) 156:144–50. 10.1001/jamadermatol.2019.3602 31851288 PMC6990853

[7] Schalock PC Dinulos JG. Mycoplasma pneumoniae-induced Stevens-Johnson syndrome without skin lesions: fact or fiction? J Am Acad Dermatol. (2005) 52:312–5. 10.1016/j.jaad.2004.07.044 15692479

[8] Santos RP Silva M Vieira AP Brito C. Mycoplasma pneumoniae- induced rash and mucositis: a recently described entity. BMJ Case Rep. (2017) 2017:bcr2017220768. 10.1136/bcr-2017-220768 28830900 PMC5623246

[9] Li HO Colantonio S Ramien ML. Treatment of Mycoplasma pneumoniae-Induced Rash and Mucositis With Cyclosporine. J Cutan Med Surg. (2019) 23:608–12. 10.1177/1203475419874444 31502864

[10] Poddighe D Bruni P. Mycoplasma pneumoniae-induced rash and mucositis (MIRM): an unusual mild skin rash associated with severe mucosal involvement. BMJ Case Rep. (2017) 2017:bcr2017220749. 10.1136/bcr-2017-220749 28551607 PMC5623314

[11] Meyer Sauteur PM. Theiler M. Mycoplasma pneumoniae-associated flagellate erythema. JAAD Case Rep. (2020) 6:1283–5. 10.1016/j.jdcr.2020.09.029 33912639 PMC8064935

[12] Bukhari EE Alotaibi FE Bugshan AS Bakheet HM Binsufayan SM Alsaadi MM. Mycoplasma pneumoniae-associated mucositis syndrome: A rare and clinically challenging disease in a Saudi child. J Taibah Univ Med Sci. (2017) 12:356–9. 10.1016/j.jtumed.2016.12.00 31435263 PMC6695005

[13] Watkins LKF Olson D Diaz MH Lin X Demirjian A Benitez AJ. Epidemiology and Molecular Characteristics of Mycoplasma pneumoniae During an Outbreak of M. pneumoniae-associated Stevens-Johnson Syndrome. Pediatr Infect Dis J. (2017) 36:564–71. 10.1097/INF.0000000000001476 28060039 PMC5893500

[14] Schalock PC Dinulos JG. Mycoplasma pneumoniae–induced cutaneous disease. Int J Dermatol. (2009) 48:673–80. 10.1111/j.1365-4632.2009.04154.x 19570071

[15] Norton SA. Diagnosing Mycoplasma pneumoniae-induced rash and mucositis (MIRM) in the emergency room. J Am Acad Dermatol. (2015) 73:e67. 10.1016/j.jaad.2015.03.060 26184002

[16] Gandelman JS Kim EY Grzegorczyk AM Zejnullahu K Edson RS. Mycoplasma pneumoniae-Induced Rash and Mucositis in a Previously Healthy Man: a case report and brief review of the literature. Open Forum Infect Dis. (2020) 7:ofaa437. 10.1093/ofid/ofaa437 33094121 PMC7568429

[17] Song H Huang JT Tan JK. Mycoplasma-Induced Rash and Mucositis in a Father and Son: A Case Report. Pediatr Infect Dis J. (2018) 37:e205–6. 10.1097/INF.0000000000001881 29278609

[18] Canavan TN Mathes EF Frieden IJ. Shinkai K. Reply to: “Diagnosing Mycoplasma pneumoniaee-induced rash and mucositis (MIRM) in the emergency room”. J Am Acad Dermatol. (2015) 73:e69. 10.1016/j.jaad.2015.04.046 26184003

[19] Zhu Z Zhang T Guo W Ling Y Tian J Xu Y. Clinical characteristics of refractory mycoplasma pneumoniae pneumoniae in children treated with glucocorticoid pulse therapy. BMC Infect Dis. (2021) 21:126. 10.1186/s12879-021-05830-4 33509121 PMC7844890

[20] Poddighe D Comi EV Brambilla I Licari A Bruni P Marseglia GL. Increased Total Serum Immunoglobulin E in Children Developing Mycoplasma pneumoniae-related Extra-pulmonary diseases. Iran J Allergy Asthma Immunol. (2018) 17:490–6. 10.18502/ijaai.v17i5.307 30518191

[21] Kunimi Y Hirata Y Aihara M Yamane Y Ikezawa Z. Statistical analysis of Stevens-Johnson syndrome caused by Mycoplasma pneumoniae infection in Japan. Allergol Int. (2011) 60:525–32. 10.2332/allergolint.11-OA-0309 22113160

[22] Mayor-Ibarguren A Feito-Rodriguez M González-Ramos J Del Rosal-Rabes T González-Sainz FJ Sánchez-Orta A. Mucositis secondary to chlamydia pneumoniae infection: expanding the mycoplasma pneumoniae-induced rash and mucositis concept. Pediatr Dermatol. (2017) 34:465–72. 10.1111/pde.13140 28568680

[23] Chaudhry R Ghosh A Chandolia A. Pathogenesis of Mycoplasma pneumoniae: an update. Indian J Med Microbiol. (2016) 34:7–16. 10.4103/0255-0857.174112 26776112

[24] Mazori DR Nagarajan S Glick SA. Recurrent reactive infectious mucocutaneous eruption (RIME): Insights from a child with three episodes. Pediatr Dermatol. (2020) 37:545–7. 10.1111/pde.14142 32172537

[25] Hyde TB Gilbert M Schwartz SB Zell ER Watt JP Thacker WL. Azithromycin prophylaxis during a hospital outbreak of Mycoplasma pneumoniae pneumoniae. J Infect Dis. (2001) 183:907–12. 10.1086/319258 11237807

[26] Poddighe D. Mycoplasma pneumoniae-related extra-pulmonary diseases and antimicrobial therapy. J Microbiol Immunol Infect. (2020) 53:188–9. 10.1016/j.jmii.2019.04.011 31153829

[27] Martinez AE Atherton DJ. High-dose systemic corticosteroids can arrest recurrences of severe mucocutaneous erythema multiforme. Pediatr Dermatol. (2000) 17:87–90. 10.1046/j.1525-1470.2000.01720.x 10792793

[28] Metry DW Jung P Levy ML. Use of intravenous immunoglobulin in children with stevens-johnson syndrome and toxic epidermal necrolysis: seven cases and review of the literature. Pediatrics. (2003) 112:1430–6. 10.1542/peds.112.6.1430 14654625

[29] Kim HS Sol IS Li D Choi M Choi YJ Lee KS. Efficacy of glucocorticoids for the treatment of macrolide refractory mycoplasma pneumoniae in children: Meta-analysis of randomized controlled trials. BMC Pulm Med. (2019) 19:251. 10.1186/s12890-019-0990-8 31852460 PMC6921474

[30] Bressan S Mion T Andreola B Bisogno G Da Dalt L. Severe Mycoplasma pneumoniae associated mucositis treated with immunoglobulins. Acta Paediatr. (2011) 100:e238–40. 10.1111/j.1651-2227.2011.02342.x 21535132

[31] Zipitis CS Thalange N. Intravenous immunoglobulins for the management of Stevens-Johnson syndrome with minimal skin manifestations. Eur J Pediatr. (2007) 166:585–8. 10.1007/s00431-006-0287-9 17008995

